# Unsupervised neural network for single cell Multi-omics INTegration (UMINT): an application to health and disease

**DOI:** 10.3389/fmolb.2023.1184748

**Published:** 2023-05-24

**Authors:** Chayan Maitra, Dibyendu B. Seal, Vivek Das, Rajat K. De

**Affiliations:** ^1^ Machine Intelligence Unit, Indian Statistical Institute, Kolkata, India; ^2^ Tatras Data Services Pvt. Ltd., New Delhi, India; ^3^ Novo Nordisk A/S, Maløv, Denmark

**Keywords:** single cell analysis, deep neural networks, unsupervised learning, CITE-seq, ATAC-seq, rare disease, multi-omics integration

## Abstract

Multi-omics studies have enabled us to understand the mechanistic drivers behind complex disease states and progressions, thereby providing novel and actionable biological insights into health status. However, integrating data from multiple modalities is challenging due to high dimensionality and diverse nature of data, and noise associated with each platform. Sparsity in data, non-overlapping features and technical batch effects make the task of learning more complicated. Conventional machine learning (ML) tools are not quite effective against such data integration hazards due to their simplistic nature with less capacity. In addition, existing methods for single cell multi-omics integration are computationally expensive. Therefore, in this work, we have introduced a novel Unsupervised neural network for single cell Multi-omics INTegration (UMINT). UMINT serves as a promising model for integrating variable number of single cell omics layers with high dimensions. It has a light-weight architecture with substantially reduced number of parameters. The proposed model is capable of learning a latent low-dimensional embedding that can extract useful features from the data facilitating further downstream analyses. UMINT has been applied to integrate healthy and disease CITE-seq (paired RNA and surface proteins) datasets including a rare disease Mucosa-Associated Lymphoid Tissue (MALT) tumor. It has been benchmarked against existing state-of-the-art methods for single cell multi-omics integration. Furthermore, UMINT is capable of integrating paired single cell gene expression and ATAC-seq (Transposase-Accessible Chromatin) assays as well.

## 1 Introduction

Recent advancements in single cell technologies have provided unprecedented opportunities in analysis of omics data. This allows researchers to probe biological functions at the cellular level while studying embryonic development, immune system or cancer (Griffiths et al., 2018; Papalexi and Satija, 2018; Wills and Mead, 2015). Existing technologies include DROP-seq (Macosko et al., 2015), SMART-seq2 Picelli et al. (2013) and 10x Genomics, which allow measuring mRNA expressions at single cell resolution (scRNA-seq). Most recently, technologies have further scaled up to produce data assays from multiple modalities. This has provided several views of the same cell of interest, thereby refining our definitions of the cellular identity. Multi-omics studies provide better understanding of the underlying biological mechanisms active during disease growth and progression, which were otherwise hidden due to the inclusion of a single omics. They also help us understand the effect of one omics layer on the other (Seal et al., 2020). A few such methods include CITE-seq (Stoeckius et al., 2017) and REAP-seq Peterson et al. (2017) which enable paired measurement of RNA and cell surface proteins. ATAC-seq (Buenrostro et al., 2015) measures chromatin accessibility while other methods like, SNARE-seq (Chen et al., 2019), sci-CAR (Cao et al., 2018) and SHARE-seq Clyde (2021) measure paired gene expression and chromatin accessibility. ScNMT (Clark et al., 2018), on the other hand, integrates single cell chromatin accessibility, DNA methylation and transcriptomics data. However, integration of various modalities of data does not come without its challenges (Lähnemann et al., 2020). High data dimension, sensitivity associated with each platform, zero-inflation due to dropouts (Jiang et al., 2022), and technical batch effects (Luecken et al., 2022) account for the stochasticity and noise in the data. An appropriate organization and analysis of these multi-modal datasets involve clever way of their integration and demand efficient computing paradigm.

At present, several methods exist that can perform the task of integration of single cell omics modalities. Seuratv3 (Stuart et al., 2019) can integrate various single cell omics datasets including RNA-seq, protein expression, chromatin and spatial data, and transfer information between them. Seuratv4 (Hao et al., 2021) provides multi-modal single cell analysis using “weighted nearest neighbor” method. MOFA+ (Argelaguet et al., 2020), based on Bayesian Group Factor Analysis, is another method that generates a low-dimensional representation of the data by integrating two or more omics among gene expression, DNA methylation and chromatin data. In recent years, several neural network-based methods have been developed for the task of single cell multi-omics integration. One such method, GLUE (Cao and Gao, 2022), integrates unpaired samples from single cell multi-omics data and also predicts regulatory interactions. Other methods, like scJoint (Lin et al., 2022) and scMVP (Li et al., 2022), can integrate scRNA-seq and scATAC-seq data. The former is a semi-supervised framework which allows label transfer and joint visualization, while the latter extracts a latent representation from the integrated data using a modified variational autoencoder model. TotalVI (Gayoso et al., 2021), on the other hand, uses an encoder function to learn a joint representation of the data and Bayesian inference to build a latent embedding from single cell RNA and protein expressions. Multigrate (Lotfollahi et al., 2022) develops an alternative pipeline for integrating CITE-seq and single cell ATAC-RNA data for both paired and unpaired samples. It has been used to map multimodal queries to reference atlases and impute missing values. Other standard omics integration methods include UINMF (Kriebel and Welch, 2022), MUON (Bredikhin et al., 2022), scMOC (Eltager et al., 2021) and SIMBA (Chen et al., 2021). A comprehensive review of major single cell multi-omics integration methods can be found in (Stanojevic et al., 2022).

With single cell multi-omics analysis, we can now comprehend the mechanisms underlying complex disease states and progressions at a cellular resolution. It has provided us multiple views of the same patient and cognizance into the individual’s health status. Some diseases, though being rare (often referred to as a rare disease (RD)), cumulatively affect quite a substantial percentage of patients. Overall, there are more than 7,000 variants of RDs. RDs affect patients’ and their families’ quality of life, and have significant societal impact. Due to the rarity of each RD, it is extremely difficult to properly diagnose and treat these individuals, as well as engage them into research to upgrade therapies. With the advancements in omics technologies, molecular understanding of RDs has improved over time leading to their rapid diagnosis. To combine multi-omics data from various technologies, Artificial Intelligence (AI)-based integration techniques are, nevertheless, becoming more and more necessary. Deep learning (DL) methodologies to integrate and query data from several heterogeneous sources may also be utilised to dramatically accelerate the discovery of efficient RD therapies (Bottini et al., 2021). A detailed review (Lee et al., 2022) exploring 332 articles on the application of DL on RDs indicate the rising demand for the use of DL for advancements in diagnosis and therapeutics of RDs.

There are, however, challenges to be addressed while using DL for multi-omics analysis for health and disease. Although different omics measurements are recorded against the same set of cells, they encode different features related to the underlying transcriptional states and activities. Existing methods to handle these datasets are not always capable of extracting features relevant to a biologically significant problem, including cell-type classification/clustering, biomarker identification, disease prediction and drug discovery. Furthermore, sparsity and noise in the data along with differences in platforms producing such high-dimensional datasets and the presence of batch effects add to the complexity of analysis (Lance et al., 2022). An inherent feature of multi-omics data in concern is its complex, non-linear, layered structure. The architecture of a deep neural network also resembles such layered non-linearity. The output from each layer is multiplied by its weight vector to compute the weighted sum, and a non-linear function is then applied over the weighted sum for each node in the layer. The non-linear output is then passed on to the next layer. Thus, deep learning models facilitate learning complex features in an unsupervised manner. However, existing neural network-based methods for single cell multi-omics integration are computationally expensive since they involve substantial amount of parameter training. Further, even though pre-processing of single cell data involves steps that may include scaling/normalization using a specific data distribution, methods for integration of such pre-processed data should be free from making assumptions about data distribution, which is not the case with most of the existing integration models.

All these problems discussed above have encouraged us to develop a robust integration method for single cell multi-omics integration that can be applied to health and disease analysis. Hence, in this work, we have introduced a novel Unsupervised neural network for single cell Multi-omics INTegration (UMINT). UMINT is competent enough to integrate different single cell omics layers of high dimensions with ease. It produces a latent low-dimensional embedding that can extract relevant features from the data, which facilitate further analyses. It can also reconstruct the data with high accuracy. Further, UMINT does not make assumptions about the distribution of data, and can integrate a variable number of omics modalities. In addition, UMINT owns a light-weight architecture and is thus computationally far less expensive than some of the existing unsupervised neural network based methods (like those based on autoencoder networks), used for single cell multi-omics integration. The performance of UMINT has been demonstrated on multiple publicly available healthy and disease datasets. These comprise four CITE-seq datasets, one of which contains cells from MALT tumor, a rare variant of malignant lymphoma. We have benchmarked the results against several existing state-of-the-art algorithms used for single cell multi-omics integration, which fall under different categories. In-silico experimental results compare favourably for UMINT against these state-of-the-art methods. Additionally, the performance of UMINT has been validated through integration of an auxiliary multimodal single cell paired gene expression and ATAC-seq (chromatin accessibility) data. Finally, as a further extension to this work, UMINT has been used to integrate bulk multi-omics data with more than two omics layers, which it has been able to execute with ease. Thus, UMINT’s ability to integrate widely heterogenous omics data (CITE-seq, paired RNA-seq and ATAC-seq) with varying number of omics layers boosts its utility as a powerful integration model and makes it completely fit in with the *status quo*.

The remaining part of this article has been organized as follows. Section 2 explains the methodology behind the proposed single cell multi-omics integration technique, called UMINT. Section 3 describes *in silico* experimental results obtained on different datasets used in this work and provides a detailed comparison against other existing methods for single cell multi-omics integration. In Section 4, the strengths and limitations of the proposed method are discussed along with concluding remarks.

## 2 Methodology

**FIGURE 1:**
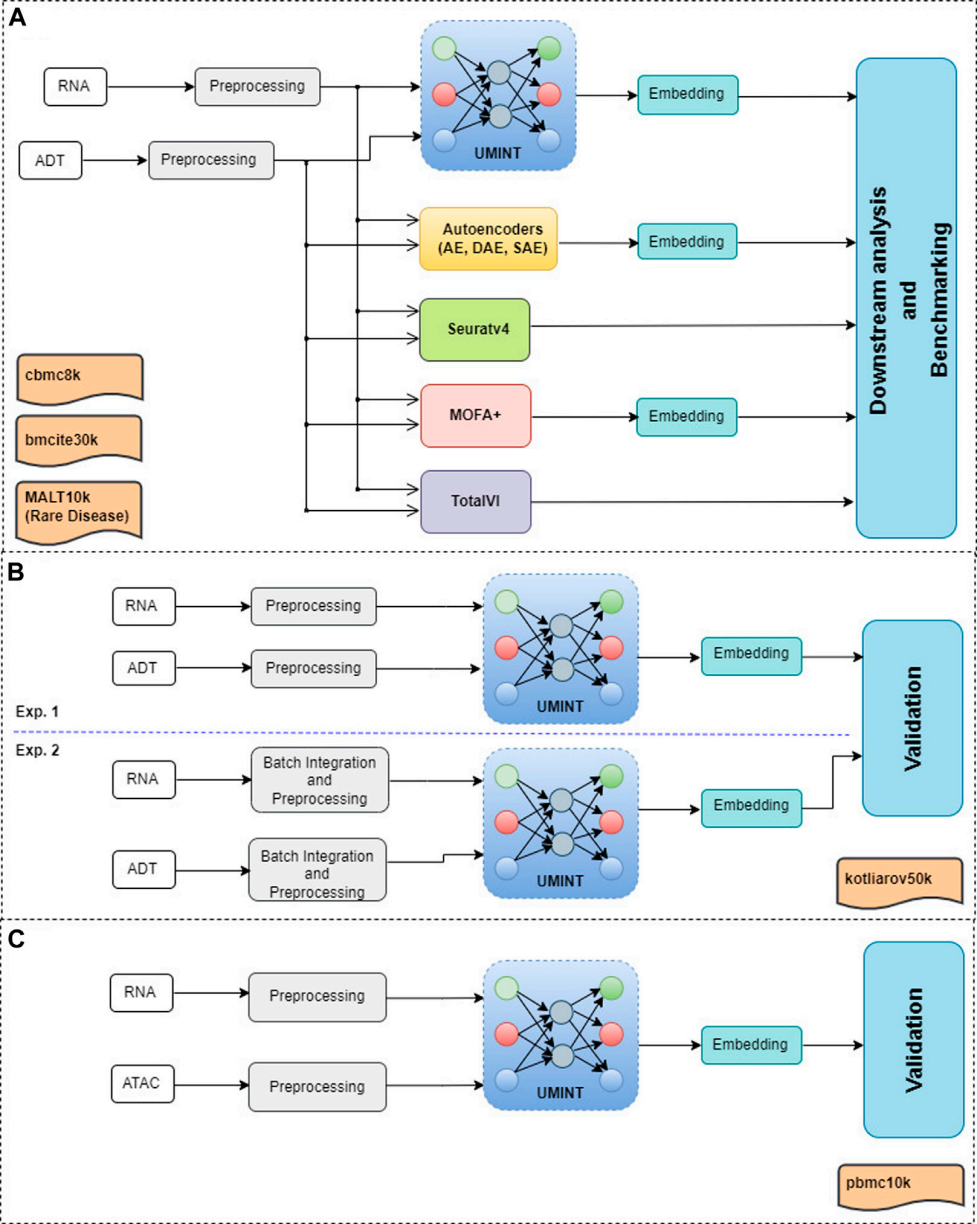
A graphical abstract showing the overall workflow of the methods used for evaluation and benchmarking of the proposed method for single cell multi-omics integration, called UMINT. Panel **(A)** shows the primary experiments conducted in this work, as described in Section 3.1 and Section 3.2. Each modalities in *cbmc*8*k*, *bmcite*30*k* and *MALT*10*k* have first been preprocessed using Seuratv4. The preprocessed datasets have been fed as input to UMINT, Autoencoder-based methods (AE, SAE and DAE), Seuratv4, MOFA+ and TotalVI. Seuratv4 and TotalVI are capable of producing a latent low-dimensional embedding and subsequently find cell clusters. The embedding produced by UMINT, AE-based methods and MOFA + have been subjected to k-means and hierarchical clustering. The clustering performance of all the methods have then been compared. Panel **(B)** shows the experiments conducted for *kotliarov*50*k*, where preprocessed data from each modality without batch integration (Exp. 1) and with batch-integration (Exp. 2) done using Seuratv4 SCTransform () have been separately fed as input to UMINT, and the clustering performance on the embeddings generated by UMINT in both these cases have been compared, as explained in Section 3.3. Panel **(C)** depicts the experiments carried out on *pbmc*10*k*, where the two modalities (RNA and ATAC) have been preprocessed using MUON and the integrated embedding produced by UMINT has been assessed for its clustering performance, as explained in Section 3.4.

This section describes the datasets used in the experiments, the methodology used for data pre-processing and the proposed neural network model, called UMINT, for single cell multi-omics integration. Figure 1 shows a graphical abstract illustrating the overall workflow of the methods used for single cell multi-omics integration, and the procedures conducted for preprocessing, embedding, validation and benchmarking performed in this work.

### 2.1 Data acquisition and pre-processing

Initially, four publicly available CITE-seq datasets, viz., *cbmc*8*k* (Stoeckius et al., 2017), *MALT*10*k* (Li et al., 2021), *bmcite*30*k* (Stuart et al., 2019) and *kotliarov*50*k* (Kotliarov et al., 2020), have been used in this work. *MALT*10*k* dataset consists of cells from a MALT tumor, a rare kind of malignant lymphoma (Oh et al., 2006). The datasets have been downloaded as count matrices and pre-processed via Seuratv4 (Hao et al., 2021). For scRNA-seq part in these datasets, we have normalized them by library size to sum up to 10,000, applied a logarithmic transformation, extracted highly variable genes, and finally scaled them linearly (with default parameters). The protein expressions/antibody-derived tag (ADT) datasets have been normalized using the centered log-ratio transformation (Stoeckius et al., 2017). Three proteins, viz., *CCR*5, *CCR*7 and *CD*10, have been removed from *cbmc*8*k* dataset due to poor abundance. The first three datasets, viz., *cbmc*8*k*, *MALT*10*k* and *bmcite*30*k*, have been used to evaluate the performance of UMINT and compare the results against other state-of-the-art algorithms. The fourth dataset, viz., *kotliarov*50*k*, contains filtered cells with highly variable genes only. It has been preprocessed via Seuratv4 and used to assess other performance criteria of the proposed methodology. Another auxilliary single cell multimodal dataset, downloaded from 10x Genomics, has been used at a later stage of the work. It contains paired ATAC and gene expression data from human PBMCs with granulocytes removed through cell sorting (processed with ARC 1.0.0 pipeline). It has been preprocessed via MUON (Bredikhin et al., 2022), and used to evaluate the performance of UMINT on paired RNA-seq and ATAC-seq data. The summary of the single cell multi-omics datasets used in this work have been listed in Table 1.

**TABLE 1:** Summary of datasets used for evaluation of UMINT.

Dataset	Description	#Cells	#RNAs	#ADTs/#Peaks	Batches present	Healthy/Disease	Source
*cbmc*8*k* (CITE-seq)	scRNAseq and antibody sequencing of CBMCs	8,617	20,501	13	No	Healthy	Stoeckius et al. (2017)
*MALT*10*k* (CITE-seq)	Cells from a dissociated Extranodal Marginal Zone B-Cell Tumour (MALT) stained with TotalSeq-B antibodies	8,412	33,538	17	No	Rare disease	Li et al. (2021)
*bmcite*30*k* (CITE-seq)	scRNA-seq profiles measured alongside a panel of antibodies from bone marrow	30,672	17,009	25	Yes	Healthy	Stuart et al. (2019)
*kotliarov*50*k* (CITE-seq)	CITE-seq profiling of 82 surface proteins and transcriptomes of 53,201 single cells from healthy high and low influenza-vaccination responders	58,654	32,738	87	Yes	Healthy	Kotliarov et al. (2020), Lotfollahi et al. (2022)
*pbmc*10*k* (paired RNA-seq and ATAC-seq)	Single cell multiome ATAC and gene expression data from cryopreserved human peripheral blood mononuclear cells (PBMCs) of a healthy female donor	11,909	36,601	108,377	No	Healthy	Bredikhin et al. (2022)

### 2.2 Unsupervised neural network for single cell multi-omics INTegration (UMINT)

**FIGURE 2:**
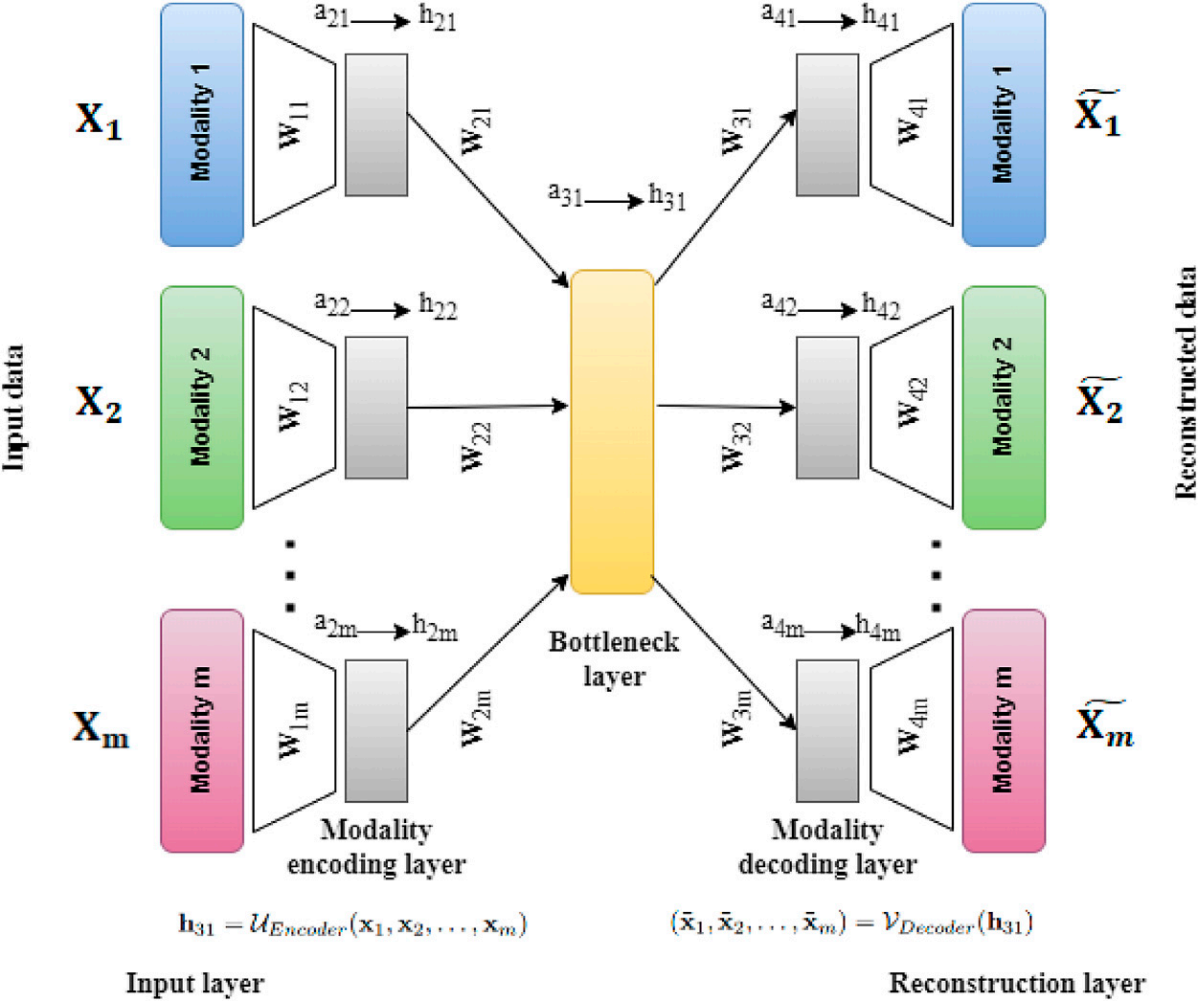
Architecture of UMINT showing propagation of input data through the network. Eq. 1 shows how the *Modality encoding layer* encodes each modality fed as input to UMINT. At the *Bottleneck layer*, integration of these modalities is performed using Eq. 2. The encoding process that combines the above mentioned steps is represented in Eq. 3. Eq. 4 shows how the *Modality decoding layer* tries to decode individual modalities and produce reconstructions. The decoding process is represented in Eq. 5. Once a reconstruction is produced, the loss is calculated using Eq. 6. The error is then propagated backwards through the network and the trainable parameters are updated accordingly.

In this work, we have developed a deep Unsupervised neural network for single cell Multi-omics INTegration (UMINT). UMINT is a non-recurrent feed-forward neural network that is efficient enough to integrate variable number of omics layers and extract a latent embedding at a reduced dimension. The network structure of UMINT represents a novel neural network architecture as shown in Figure 2.

Let **X**_1_, **X**_2_, …, **X**_*m*_ be *m* datasets corresponding to *m* different omics modalities having *n* samples (cells) each with *d*_1_, *d*_2_, …, *d*_*m*_ features (RNAs in case of gene expression data, ADTs in case of protein expression data or Peaks in case of transposase-accessible chromatin data) respectively. The UMINT architecture consists of two sub-architectures - an encoder and a decoder. The encoder accepts data from multi-omics datasets presented to the *Input layer*, transports them through one or more *Modality encoding layer*(s) and integrates them in the final layer, known as the *Bottleneck layer*. The decoder accepts the embedded output from the *Bottleneck layer*, transports them through one or more *Modality decoding layer*s and finally tries to reconstruct the original data at the *Reconstruction layer*. In this work, we have used only one layer each for modality encoding and decoding. However, UMINT may contain multiple such layers based on the requirement. In order to improve generalization capability and reduce dimension of the latent embedding, the number of neurons at the *Bottleneck layer* has been kept smaller than the number of neurons in the *Input layer*.

#### 2.2.1 Forward propagation

The *Input layer* of UMINT consists of *m* different modules, each of which accepts input from a data modality. The number of neurons in each of the modules in the *Input layer* is equal to the dimensions of the individual data modalities *d*_1_, *d*_2_, …, *d*_*m*_ respectively. In this layer, UMINT tries to find a suitable projection for each of the data modalities that may be good enough to get integrated in subsequent layers. Each module in the first *Modality encoding layer* shares a dense connection with the corresponding modules of the *Input layer*. The first *Modality encoding layer* containing *m* modules thus accepts data from *m* modules in the *Input layer* as input, and obtains *m* different projections. Let **a**_*ji*_ and **h**_*ji*_ be the input to and the output from the *ith* module in the *jth* layer respectively. Then, for the *Input* and *Modality encoding* layers, we have

$$\begin{array}{cccc} a_{1i} & =x_{i} & h_{1i} & =a_{1i}\\ a_{2i} & =W_{1i}h_{1i}+b_{1i} & h_{2i} & =ReLU\left(a_{2i}\right) \end{array}$$ (1)

where **x**_*i*_ is a sample in *ith* modality, and *ReLU*(**y**) = *max*(**0**, **y**). The term **W**_1*i*_ denotes the weights between *ith* module of the *Input layer* and *ith* module of the first *Modality encoding layer*, and **b**_1*i*_ denotes the bias terms of the nodes in *ith* *Modality encoding layer*. Finally, the outputs from the *Modality encoding layer*(s) are projected onto a lower dimensional space in the *Bottleneck layer*. The final *Modality encoding layer* and the *Bottleneck layer* are fully connected. If **W**_2*i*_ represents the weights between the *ith* module of the final *Modality encoding layer* and the *Bottleneck layer*, then we have

$$a_{31}=\overset{m}{\underset{i=1}{\sum }}\left(W_{2i}h_{2i}\right)+b_{21}\;\;h_{31}=ReLU\left(a_{31}\right)$$ (2)

where **b**_21_ denotes the bias terms of the nodes in *Bottleneck layer*. This concludes the process of encoding. The overall function of the encoder network can thus be represented as

$$h_{31}=U_{\mathrm{Encoder}}\left(x_{1},x_{2},\ldots ,x_{m}\right)$$ (3)

Reconstruction of the original data is done by the decoder network in exactly the opposite manner to that of encoding. The integrated embedding coming out of the *Bottleneck layer* is projected onto the *Modality decoding layer*(s) which consists of the same number of modules as that in the *Modality encoding layer*(s). The number of neurons in each module of the *Modality decoding layer* is identical to that used in the modules in the *Modality encoding layer*. The last layer of the decoder is the *Reconstruction layer* which tries to reconstruct the original data from the respective modules in the final *Modality decoding layer*. The process of decoding can be expressed as

$$\begin{array}{cccc} a_{4i} & =W_{3i}h_{31}+b_{3i} & h_{4i} & =ReLU\left(a_{4i}\right)\\ a_{5i} & =W_{4i}h_{4i}+b_{4i} & \;\;h_{5i} & =a_{5i}={\tilde{x}}_{i} \end{array}$$ (4)

where **W**_*ji*_ and **b**_*ji*_ represent weights and biases for the *ith* module in the *jth* layer respectively. Thus, the decoder function is given by

$$\left({\tilde{x}}_{1},{\tilde{x}}_{2},\ldots ,{\tilde{x}}_{m}\right)=V_{\mathrm{Decoder}}\left(h_{31}\right)$$ (5)

where ${\tilde{x}}_{i}$ denotes the reconstruction for **x**_*i*_.

#### 2.2.2 Objective function

In this scope of work, UMINT has been initially used to integrate scRNA-seq and single cell protein expression data. Subsequently, it has been used to integrate scRNA-seq and ATAC-seq data. Thus, for each dataset, paired RNA and ADT assays, or paired RNA and ATAC assays form the inputs to different modules of the *Input Layer* of UMINT. For each cell **x**, UMINT tries to find an optimal reconstruction $\tilde{x}$ of the input data retaining as much information as possible, thereby minimizing the reconstruction error $\left\|x-\tilde{x}\right\|$ . The reconstruction error is contributed by reconstruction loss from each modality. In order to avoid biasness arising out of number of dimensions in the input modalities, we have introduced a balancing parameter *λ*_*i*_. Additionally, in order to limit over-fitting, we have used an *L*1 regularization on the nodes’ activities to allow sparsity of nodes’ outputs and an *L*2 regularization on the weight values since *L*2 regularization tries to shift weight values towards zero. Both *L*1 and *L*2 regularizations minimize the model complexity. In this work, *L*1 and *L*2 regularizations have been controlled using regularization parameters *α* and *β* respectively. The objective function thus becomes

$$L_{\mathrm{UMINT}}=\frac{1}{n}\overset{m}{\underset{i=1}{\sum }}\lambda_{i}\|X_{i}-{\tilde{X}}_{i}{\|}^{2}+\overset{m}{\underset{i=1}{\sum }}\overset{4}{\underset{j=2}{\sum }}\left(\alpha \|h_{ji}{\|}_{1}+\beta \|W_{ji}{\|}_{2}\right)$$ (6)

Values of the regularization parameters *α* and *β* have been set to 0.0001 and 0.001 respectively, as recommended in literature (Chaudhary et al., 2018). UMINT has been trained for 25 epochs using Adam Optimizer (Kingma and Ba, 2017) with a batch size of 16 and Eq. 6 as the loss function. During the forward pass, the data is fed as input to the encoder. A lossy reconstruction of the input data is produced by the decoder at the *Reconstruction layer*. The error value is then propagated backwards, and the weights and biases are updated for a better reconstruction in the next forward pass.

### 2.3 Latent low-dimensional embedding and clustering

At the outset of this work, the proposed integration model, called UMINT, has been used to integrate RNA and protein expression data. Once trained to reconstruct the input data, UMINT is capable of learning a latent low-dimensional embedding that extracts relevant features from the integrated data. Here, we have used UMINT to extract a latent embedding of 64 dimensions. This latent embedding has been used in the subsequent step for downstream analysis in order to explore its effectiveness. We have used agglomerative hierarchical clustering and k-means clustering algorithm on this latent embedding to cluster the cell-types for each of the datasets used in the study. The performance of UMINT has then been compared against existing benchmark methods used for multi-omics integration. We have used two measures, viz., Adjusted Rand Index (ARI) and Fowlkes Mallows Index (FMI) scores, to measure the degree of agreement between the actual and predicted cell-types, for all the methods used in comparison including UMINT. The actual cell types corresponding to the ground truth data have been obtained from the corresponding source datasets mentioned in Table 1.

For two sets of cluster labels, the overlap between them is represented by a contingency table **C** = [*c*_*ij*_], where *c*_*ij*_ indicates the total number of points belonging to both *ith* cluster of the first set and *jth* cluster of the second set. ARI is an external cluster validity index, and is thus defined as

ARI=∑ijcij2−∑ipi2∑jqj2/N212∑ipi2+∑jqj2−∑ipi2∑jqj2/N2 (7)

where *p*_*i*_ = *∑*_*j*_ *c*_*ij*_, *q*_*j*_ = *∑*_*i*_ *c*_*ij*_ and *N* = *∑*_*ij*_ *c*_*ij*_ respectively. An ARI value close to 1 indicates good resemblance between two clusters. Similarly, FMI, another external evaluation index used to measure the similarity between two sets of cluster labels, is defined as

$$FMI=\frac{TP}{\sqrt{\left(TP+FP\right)\left(TP+FN\right)}}$$ (8)

where *TP*, *FP* and *FN* denotes the count of True Positives, False Positives and False Negatives respectively. The FMI score lies between 0 and 1, and a high value implies a good similarity between two clusters.

At a subsequent stage of the work, UMINT has been evaluated on another multiome dataset containing paired gene expresssion and ATAC-seq data. After preprocessing each modality and reducing them to highly variable features, UMINT has been used to extract a latent 64-dimensional embedding by integrating the RNA and ATAC assays. This latent embedding has been further subjected to agglomerative hierarchical clustering and k-means clustering. The embedding quality has been assessed using external evaluation criteria like, ARI and FMI, as explained above.

## 3 Results

UMINT has been applied on a variety of datasets containing cells from both healthy donors as well as donors with a disease, and its performance has been evaluated over multiple steps as depicted in the graphical abstract shown in Figure 1. Initially, it has been benchmarked on three CITE-seq datasets, viz., *cbmc*8*k*, *MALT*10*k* and *bmcite*30*k*, where *cbmc*8*k* and *bmcite*30*k* contain cells from healthy samples, and *MALT*10*k* contains cells from rare lymphoma (MALT). The latent embedding produced by UMINT has been first compared with that produced by Autoencoder (AE)-based architectures. Subsequently, the UMINT-generated embedding has been compared with three other state-of-the-art single cell multi-omics integration methods. Section 3.1 and Section 3.2 describe results of comparison of UMINT against AE and other state-of-the-art methods on these three datasets. Thereafter, UMINT has been further tested for its performance on another CITE-seq dataset *kotliarov*50*k* having multiple batches. For evaluating the batch correction performance of UMINT, we have performed two different experiments on *kotliarov*50*k* dataset, with and without batch integration. Finally, as an extended utility, UMINT has been used to integrate paired gene expression and ATAC-seq data. The integrated embedding generated by UMINT has then been validated through clustering techniques. Additionally, UMINT has been validated on a bulk multi-omics cancer dataset for integration and classification.

### 3.1 Comparison with autoencoder-based unsupervised neural network models

An AE network (Hinton and Salakhutdinov, 2006), which is an unsupervised neural network used for dimension reduction, being the closest resemblance to UMINT, we have first compared it with a regular AE network and its variations, viz., Denoising AE (DAE) and Sparse AE (SAE), both in terms of architectural difference and performance.

#### 3.1.1 Comparison with autoencoder-based models with respect to number of trainable parameters and execution time

Similar to an AE network, UMINT also tries to reconstruct the original input as explained in Section 2.2. However, there is a difference between the two. The input layer in AE shares a dense connection with the first hidden layer, whereas, the connections between the *Input layer* and the first *Modality encoding layer* in UMINT is not dense. This reduces the number of parameters to be trained, drastically. Although, in this work, UMINT has been used to integrate single cell RNA and protein expression data, it is quite capable of integrating any number of omics layers. Let us consider that the input to UMINT consists of data from *m* modalities having *n* samples each with *d*_1_, *d*_2_, …, *d*_*m*_ dimensions respectively. As shown in Figure 2, UMINT consists of the same number of modules in the *Modality encoding layer* as that in the *Input layer*. If the number of neurons in each of the module of this *Modality encoding layer* are *n*_1_, *n*_2_, …, *n*_*m*_ respectively, then the total number of trainable parameters (*TP*_*UMINT*_) between the *Input layer* and the *Modality encoding layer* in the encoder network becomes

$$TP_{\mathrm{UMINT}}=\overset{m}{\underset{i=1}{\sum }}d_{i}n_{i}$$ (9)

Considering an input of similar dimensions, if an AE network is employed to achieve this same task of integration, the number of trainable parameters (*TP*_*AE*_) between the input layer and the first hidden layer becomes

$$TP_{AE}=\left(\overset{m}{\underset{i=1}{\sum }}d_{i}\right)\left(\overset{m}{\underset{i=1}{\sum }}n_{i}\right)$$ (10)

Thus, the reduction in the number of trainable parameters (*TP*_*Reduction*_) in the encoder network is given by

$$TP_{\mathrm{Reduction}}=TP_{AE}-TP_{\mathrm{UMINT}}=\overset{m}{\underset{i=1}{\sum }}\left(d_{i}\overset{m}{\underset{j=1,j\neq i}{\sum }}n_{j}\right)$$ (11)

A reduction in the number of trainable parameters by the same amount is also available at the *Reconstruction layer* of the decoder network. Hence, the total reduction (*TP*_*TotalReduction*_) in the number of trainable parameters in UMINT is given by

$$TP_{\mathrm{TotalReduction}}=2\times TP_{\mathrm{Reduction}}=2\times \overset{m}{\underset{i=1}{\sum }}\left(d_{i}\overset{m}{\underset{j=1,j\neq i}{\sum }}n_{j}\right)$$ (12)

This is a massive improvement over AEs considering more than one modality of data to be integrated. UMINT network reduces to a regular AE network if a single modality is used which, however, does not serve the purpose of integration.

**FIGURE 3:**
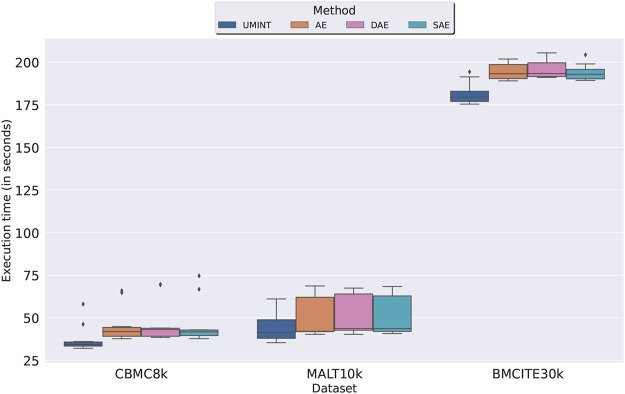
Comparison of execution time taken for single cell multi-omics data integration by UMINT with that taken for integration by different variations of AE.

We have further recorded the execution time taken by both UMINT and the different variations of AE for integration of single cell multi-omics data. This experiment has been repeated multiple times with different training and test datasets to ensure stability of results. As shown in Figure 3, we have observed that integration using UMINT has been much faster as compared to that obtained using different AE-based networks, like a regular AE, DAE and SAE. Thus, we can say that UMINT not only has a light-weight architecture than AE-based networks, but it is also computationally less expensive than them.

#### 3.1.2 Comparison with autoencoder-based models with respect to performance

**FIGURE 4:**
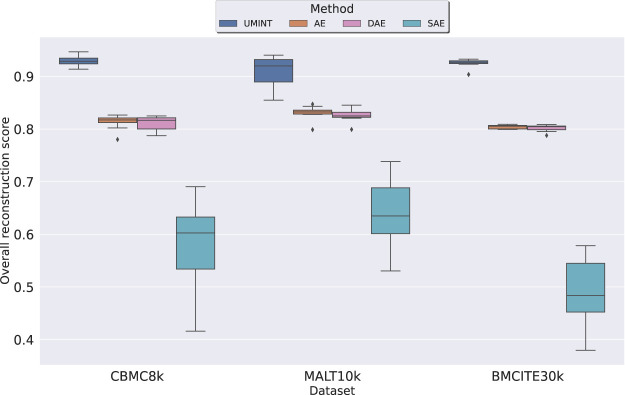
Comparison of performance of UMINT with that of a regular AE, and its variations DAE and SAE with respect to overall reconstruction of RNA and ADT modalities in *cbmc*8*k*, *MALT*10*k* and *bmcite*30*k* datasets.

Initially, the performance of UMINT has been compared with that of a standard AE and its variations (DAE, SAE) with respect to their reconstruction capability and the strength of the latent low-dimensional embeddings produced at the bottleneck layer by each network. As mentioned earlier, RNA and protein expression data form the input to different modules of the *Input Layer* of UMINT which has then been used to reconstruct the input data. On a similar note, RNA and protein expression data have been stacked together to form the input to the AE-based networks. The AE-based networks reconstruct the combined input by passing it through a series of layers. In the process, they learn to extract useful features at the bottleneck layer, where a latent embedding of 64 dimensions is produced, similar to UMINT. The models UMINT, AE, DAE and SAE have been trained keeping all hyper-parameter values identical. For each modality, the amount of correlation between the original data and its reconstruction, has then been computed for UMINT and all the AE-based models using Pearson correlation coefficient. We have then defined an Overall Reconstruction Score (ORS) to assess the reconstruction performance of UMINT against that of the AE-based models, based on the omics modalities used for integration as follows

$$ORS=\frac{1}{m}\overset{m}{\underset{i=1}{\sum }}\rho_{i}$$ (13)

where *ρ*_*i*_ is the Pearson correlation coefficient value between the pairwise distances in the original *ith* data modality and the pairwise distances in its reconstructed counterpart. As shown in Figure 4, we have observed that UMINT has outperformed all the AE-based networks with respect to overall reconstruction of the omics modalities, for all the three datasets used for evaluation, with Median Correlation Coefficient (MCC) values and corresponding *p*-values as recorded in Supplementary Table S1. All the experiments have been repeated multiple times with different training and test datasets divided in a 80 : 20 ratio. The results of the test for statistical significance thus obtained, have made us infer that UMINT is capable of producing better overall reconstructions than AE-based methods.

Thereafter, we have compared the latent low-dimensional embedding produced by UMINT with that produced by a standard AE and its variations. Once trained to create a lossy reconstruction of the input, the latent representation has been extracted from the bottleneck layer of UMINT and all AE-based models. Cell-type clustering on this latent embedding using k-means and agglomerative hierarchical clustering has been performed to validate the effectiveness of UMINT and compare it with AE-based models using ARI and FMI scores, as discussed earlier.

**FIGURE 5:**
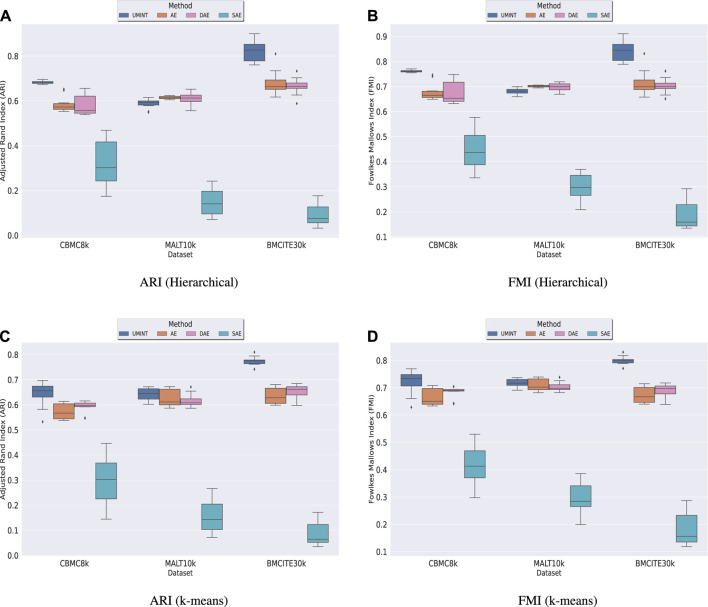
Comparison of clustering performance of UMINT against that of AE-based methods when agglomerative hierarchical clustering is used, as measured by **(A)** ARI and **(B)** FMI; **(C)** and **(D)** show clustering performance of UMINT compared to AE-based methods, as measured by ARI and FMI respectively, when k-means clustering algorithm is used.

We have observed that for *cbmc*8*k* and *bmcite*30*k* datasets, the latent representation produced by UMINT has been more representative of the cell clusters when compared with that of AE-based methods. For *MALT*10*k* dataset, when hierarchical clustering algorithm is used, UMINT embedding has produced similar ARI and FMI scores to that obtained on embedding produced by AE-based methods. This is indicated by both ARI and FMI scores as shown in Figures 5A–D. Median ARI (MARI), Median FMI (MFMI) scores along with the corresponding *p*-values obtained using UMINT and the AE-based models on these three datasets for both hierarchical and k-means clustering algorithms have been shown in Supplementary Table S2. All the experiments have been repeated multiple times with different training and test datasets to ensure stability of the results.

### 3.2 Comparison with other state-of-the-art methods for single cell multi-omics integration

**FIGURE 6:**
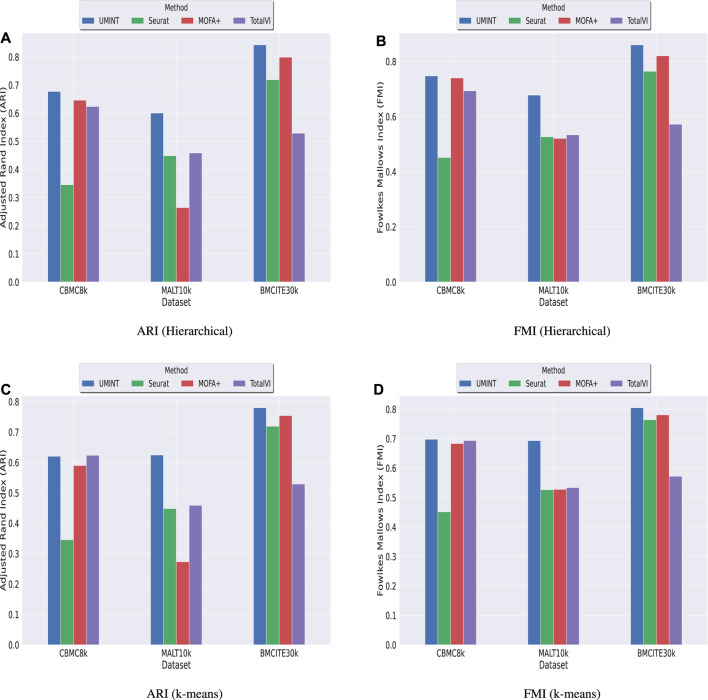
Comparison of clustering performance of UMINT against Seuratv4, MOFA+ and TotalVI when agglomerative hierarchical clustering is used, as measured by **(A)** ARI and **(B)** FMI; **(C)** and **(D)** show performance of each method, as measured by ARI and FMI respectively, when k-means algorithm is used for clustering.

Subsequently, we have compared the performance of UMINT with three other state-of-the-art methods - Seuratv4 (Hao et al., 2021), MOFA+ (Argelaguet et al., 2020) and TotalVI (Gayoso et al., 2021). We have chosen these three methods since they represent three different categories of algorithms - Graph based, Matrix factorization based and Neural network based, developed for single cell multi-omics integration (Stanojevic et al., 2022). To ensure a fair comparison between all these methods, we have followed the same preprocessing pipeline for all the datasets used for comparison. The effectiveness of UMINT has once again been demonstrated by clustering the cell-types on the latent low-dimensional embedding produced by it. It may be mentioned here that Seuratv4 is capable of producing an integrated low-dimensional representation through weighted-nearest neighbor analysis, and also find cell clusters from the integrated embedding using Louvain (Blondel et al., 2008), Leiden (Traag et al., 2019) or SLM (Waltman and Van Eck, 2013) community-detection algorithms. TotalVI, on the other hand, integrates the data through variational inferencing and autoencoding, and uses the standard Scanpy (Wolf et al., 2018) pipeline for clustering on the latent embedding. MOFA+, however, only produces a low-dimensional representation of the integrated data, as in the case of UMINT. The methods used in this work for comparison have been compared theoretically in Table 2. Thus, for Seuratv4 and TotalVI, we have extracted the cluster labels, while for MOFA+, we have extracted the factors representing the low-dimensional embedding and used hierarchical and k-means clustering on the same, similar to UMINT, as illustrated in the graphical abstract shown in Figure 1. Interestingly, UMINT has outperformed all three methods in terms of ARI and FMI scores, validated both by hierarchical and k-means clustering. Figure 6 shows the average ARI and FMI scores obtained using UMINT plotted against the scores obtained by the three benchmark methods.

**TABLE 2:** A theoretical comparison between UMINT and other methods used for comparison.

Method	Methodology	Produces latent embedding (Yes/No)	Support cell-type clustering (Yes/No)	Omics integration supported for	Makes assumption about data distribution (Yes/No)	Can reconstruct original data (Yes/No)
UMINT	Neural network based	Yes	No	Both single cell and bulk	No	Yes
Autoencoder	Neural network based	Yes	No	Both single cell and bulk	No	Yes
Seuratv4	Graph based	Yes	Yes, via Louvain, Leiden and SLM	Single cell only	No	No
MOFA+	Matrix factorization based	Yes	No	Both single cell and bulk	Yes	Yes
TotalVI	Neural network based	Yes	No (recommends using Scanpy)	Single cell only	Yes	Yes

### 3.3 Performance of UMINT on multi-batch datasets

**FIGURE 7:**
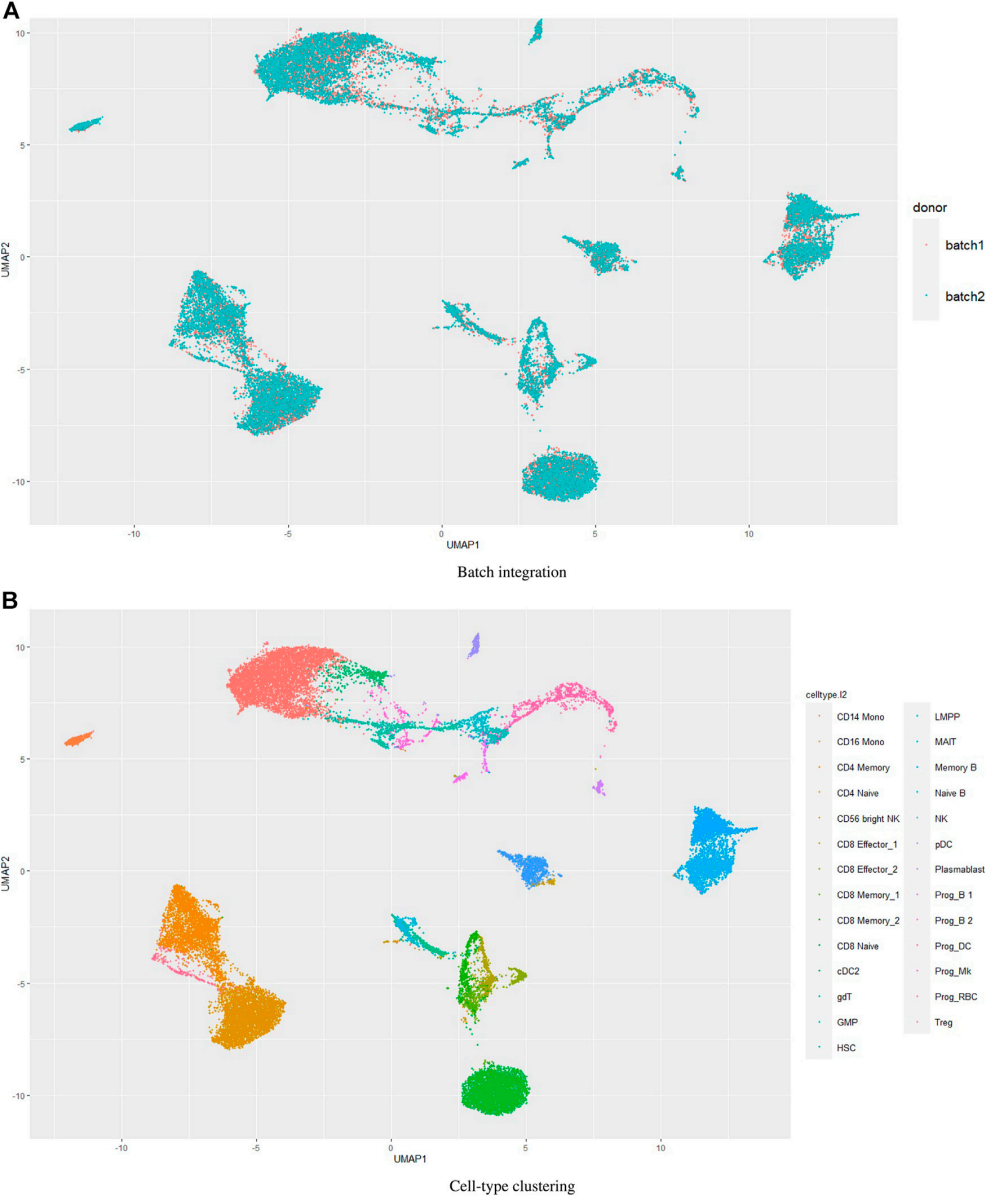
Performance of UMINT on *bmcite*30*k* dataset with respect to **(A)** batch integration, **(B)** cell-type clustering.

Batch effects in single cell datasets pose great challenges in data integration and compromises the results (Haghverdi et al., 2018; Tran et al., 2020). We wondered how UMINT would perform when there are batches in the data. The dataset *bmcite*30*k* used in this work contains two batches. However, we have not performed batch integration on this dataset. Figures 4–6 show the performance of UMINT on *bmcite*30*k* dataset when no batch integration has been performed. We have further observed that batches present in the *bmcite*30*k* projection by UMINT have been well integrated and are thus inseparable. Additionally, cell clusters obtained on UMINT projection are cohesive and well separated too. Thus, we can say that besides cell-type clustering, UMINT may have the potential to integrate batches in data efficiently, as shown in Figure 7. However, validation on a single dataset might not establish the strength of UMINT in terms of batch correction since the *bmcite*30*k* dataset itself may not have strong batch effects.

**FIGURE 8:**
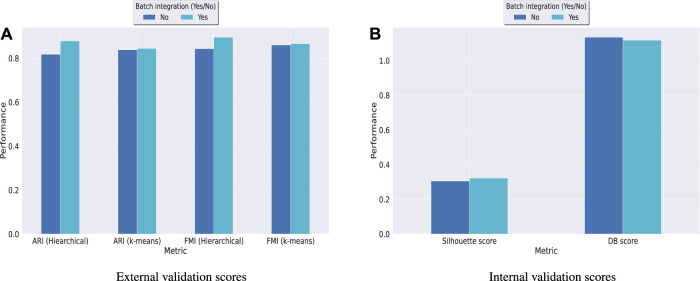
Clustering performance of UMINT-generated embeddings obtained from RNA and ADT data with and without batch integration on the *kotliarov*50*k* dataset as measured by **(A)** external validity indices and **(B)** internal validity indices.

**FIGURE 9:**
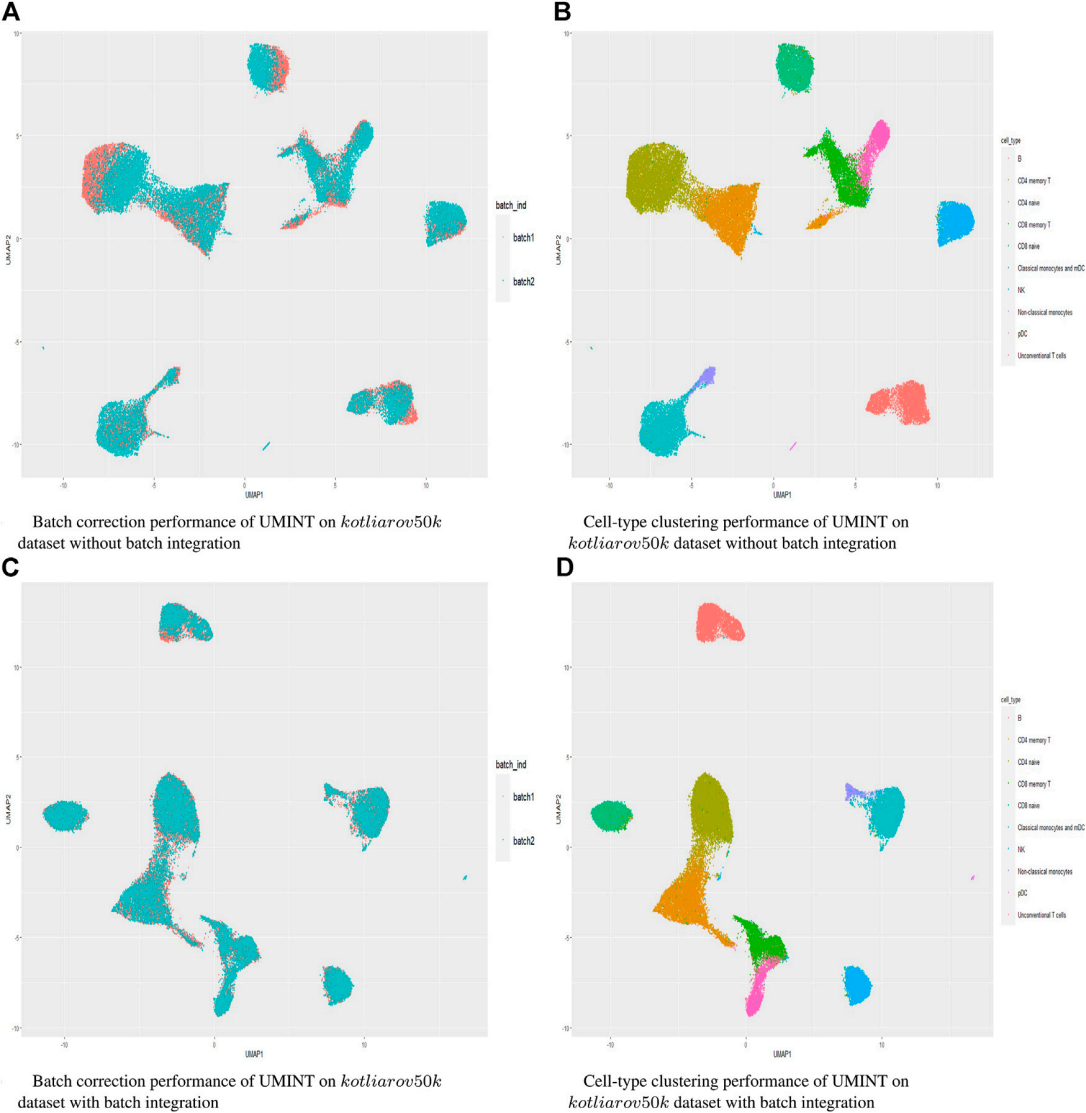
**(A)** and **(B)** show batch correction and clustering performance of UMINT on the *kotliarov*50*k* dataset without batch integration; **(C)** and **(D)** show similar results on the *kotliarov*50*k* dataset with batch integration.

Hence, in order to reinforce our findings, we have performed a few more experiments on another dataset *kotliarov*50*k*. This dataset, collected from (Lotfollahi et al., 2022), contains filtered data for 52,117 cells with highly variable genes (3,999), and two batches of RNA and protein expressions each. Moreover, it contains expression values for 87 proteins, a lot more than the three other datasets used in this work. We have first integrated batches using Seuratv4 SCTransform () (Hao et al., 2021) module with default parameters and fed the batch integrated RNA and ADT datasets to UMINT. The low-dimensional embedding produced by UMINT has then been evaluated for clustering and batch integration performance. In another experiment, we have fed the preprocessed RNA and ADT datasets (without batch integration) into UMINT, and evaluated the low-dimensional embedding produced by it for clustering and batch integration performance too. Apart from two external validity indices, we have used two internal validity indices - silhouette coefficient (Rousseeuw, 1987) and Davies Bouldin (DB) index (Davies and Bouldin, 1979) to measure the clustering performance of the UMINT-generated embeddings on the omics data with and without batch integration. Interestingly, we have observed that when the UMINT embedding has been generated from the RNA and ADT data without batch integration, the ARI, FMI, Silhouette and DB scores achieved have been quite close to those achieved when UMINT embedding has been generated from batch integrated RNA and ADT data, as shown in Figure 8. Thus, it is clear that even without batch integration, UMINT can extract most relevant features from the data that can act as input to further downstream investigations. However, the UMINT-generated embedding obtained on batch integrated data has shown better batch correction performance than that obtained on data without batch integration. From Figures 9A, C, it can be observed that batches in *kotliarov*50*k* data remain separable if batch integration is not performed on the dataset explicitly. This explains why performance on *kotliarov*50*k* dataset without explicit batch correction is not as good as that on the same dataset with batch correction. Thus, there is further scope of improvement for UMINT in terms of batch correction performance. Figures 9B, D show cell-type clustering performance of UMINT on the *kotliarov*50*k* dataset, without and with batch integration respectively.

### 3.4 Performance of UMINT on paired RNA-seq and ATAC-seq data

**FIGURE 10:**
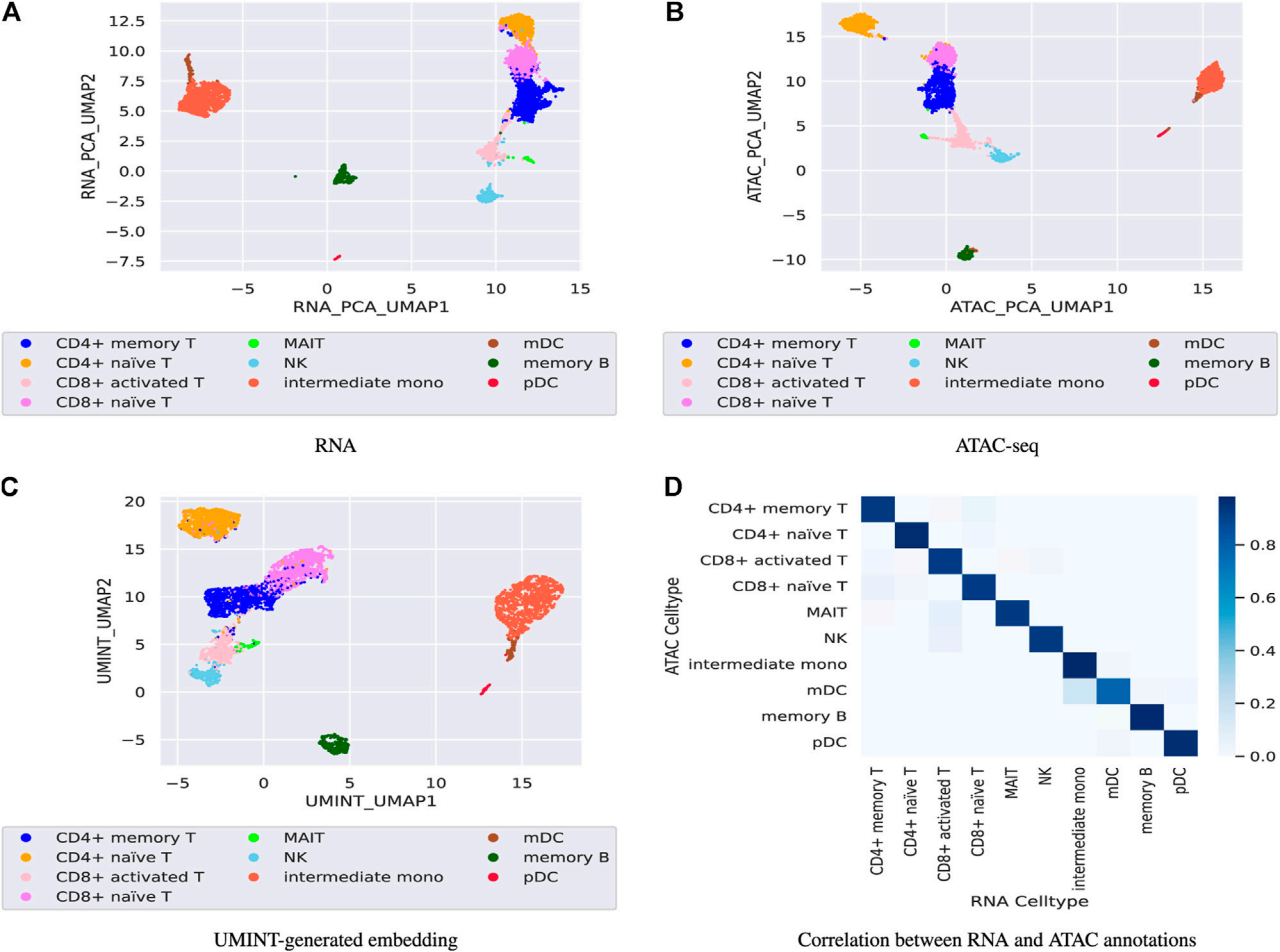
Figures **(A)**, **(B)** show UMAP projections on the individual RNA-seq and ATAC-seq data respectively after PCA-based dimension reduction while, Figure **(C)** show UMAP projection on the UMINT-generated latent embedding of *pbmc*10*k* multiome dataset. Figure **(D)** shows the correlation between the RNA and ATAC annotations.

Finally, the performance of UMINT has been assessed on another multiome dataset containing paired gene expression and ATAC-seq assays. This *pbmc*10*k* dataset has been first preprocessed via MUON (Bredikhin et al., 2022) and reduced to highly variable features only. These reduced datasets have been further processed to match the cells in the two modalities. The two paired assays, RNA and ATAC, have then been fed as input to UMINT, which has successfully extracted a latent low-dimensional embedding out of the integrated data. In order to validate the embedding quality, we have used both hierarchical and k-means clustering techniques on the UMINT-generated embedding and measured the clustering performance using ARI and FMI scores. The average scores over multiple runs of this experiment has been reported in Table 3, which shows that the UMINT-generated embedding has been pretty efficient in clustering the cell-types. The UMAP-projections on the individual modalities (after PCA-based dimension reduction) and on the embedding produced by UMINT have been illustrated in Figures 10A–C respectively. Cell-type annotation for the UMAP plot on the UMINT-generated embedding has been performed using the RNA-annotations since the ATAC annotations highly correlate to the RNA annotations as shown in Figure 10D. Thus, we can say that besides CITE-seq data, UMINT is competent enough to integrate paired RNA-seq and ATAC-seq assays too.

**TABLE 3:** ARI and FMI scores obtained on applying k-means and hierarchical clustering on the UMINT-generated latent embedding of *pbmc*10*k* multiome dataset.

k-means		Hierarchical	
**ARI**	**FMI**	**ARI**	**FMI**
0.69	0.74	0.73	0.77

### 3.5 Performance of UMINT on bulk multi-omics data

As an extension to this work, in order to support the claim that UMINT can integrate a variable number of omics layers, we have further assessed UMINT for its integration performance on bulk expression datasets with more than two modalities. TCGA multi-omics data for Liver Hepatocellular Carcinoma (LIHC) from TCGA portal (now relocated to Genomic Data Commons https://gdc.cancer.gov/), have been used for this purpose. Pre-processed datasets have been collected from our earlier work (Seal et al., 2020) in which we tried to estimate gene expression surrogates from genetic and epigenetic features through a DL pipeline. This dataset contains three omics layers - DNA methylation (DNAm), Copy Number Variation (CNV) and RNA-seq. It contains 404 paired samples out of which 359 are cancer and 45 are normal. The procedures conducted in this separate experiment and their corresponding results have been described in Supplementary Section S2.

## 4 Discussion and conclusion

In this work, we have introduced a novel deep Unsupervised neural network for single cell Multi-omics INTegration (UMINT). We have used UMINT to integrate heterogenous single cell omics modalities and extract meaningful projections at reduced dimensions. These features have been further used for clustering. The effectiveness of UMINT has been first demonstrated on four publicly available CITE-seq datasets, and compared on three of them with some other state-of-the-art algorithms used for single cell multi-omics integration. One of the three datasets used for benchmarking corresponds to MALT rare disease, which establishes the applicability of UMINT on rare diseases as well. Thereafter, the performance of UMINT has been assessed on an auxiliary dataset containing paired gene expression and ATAC-seq assays.

The strengths or advantages of UMINT are many-fold. UMINT-generated latent embedding has been proved to produce better clustering than that generated using AE-based methods. UMINT has a light-weight architecture in terms of the number of trainable parameters. Even then, UMINT-generated reconstruction has been better than that produced by AE-based methods. When evaluated against other state-of-the-art algorithms, UMINT has displayed superior performance over every other method used for comparison, across most of the evaluation criteria on all the three CITE-seq datasets. The fact that UMINT can integrate both CITE-seq, and paired RNA-seq and ATAC-seq data fortifies its strength over other existing single cell multi-omics integration methods. Moreover, UMINT does not make assumptions about the underlying data distribution, thus making it more robust. Finally, UMINT has been found to be competent enough to integrate bulk multi-omics datasets too. It has been able to produce better reconstructions for bulk omics data than that obtained using a standard AE. UMINT-extracted features from bulk multi-omics data, have also shown superior classification of tumour and normal samples. Integration of both single cell and bulk multi-omics datasets imply that UMINT supports integration of widely heterogenous and varying number of omics modalities. Very few such integration methods exist that can efficiently integrate features from both single cell and bulk multi-omics, and can handle variable number of omics layers. UMINT’s capacity to embed features from healthy and disease omics (including a rare disease) also demonstrates its applicability across varying health conditions.

UMINT, however, is susceptible to batch effects to some extent. It has been able to correct batches for *bmcite*30*k* dataset well (though the inherent batch effect in this dataset is subject to further investigations), while for *kotliarov*50*k* data, integration has been compromised by a tolerable amount due to batch effect. Further, there is a huge imbalance of features in CITE-seq, and the paired RNA-seq and ATAC-seq data. Despite this imbalance, the overall embedding produced by UMINT remains unaffected. In the current scope of work, we have not explored the integration of other high throughput omics modalities like spatial transcriptomics. Sequencing-based spatial transcriptomics data like 10x Visium are still not available at a single cell resolution. They are at spot level which may contain around 10–30 cells per spot on an average which hinders pairing of input samples (cells and spots). Presently, deconvolution methods are still a better choice for interrogating spatial trancriptomics with single cell transcriptomics. We have also not optimized the model at this stage for image-based data, hence multi-omics spatial data, like NanoString GeoMx, MERSCOPE using MERFISH technology, cannot be still used for integration. Inclusion of such omics layer(s) will need inclusion of Convolutional Neural Network (CNN)-based DL models into the existing UMINT architecture. This would further allow us to better understand the overall contribution of each omics layer at a single cell and spatial level to decipher regulatory systems biology on top of scRNA-seq, scATAC-seq and protein expression data with a spatial location. The potentiality of UMINT to select features from each of the input modalities has also not been explored in the current scope of work. Instead, UMINT has been used to extract relevant features from the integrated data at a low dimension. All these remain as a future extension and a scope for improvement for UMINT to identify key molecular anchors in development and disease biology.

Nevertheless, UMINT can capture better variability among high-dimensional datasets and produce robust low-dimensional embedding which can significantly assist further downstream analyses. A reduction in the number of trainable parameters also makes UMINT far less computationally expensive than existing neural network models based on AEs. Thus, we are able to provide a robust and efficient unsupervised deep learning model for single cell multi-omics integration.

## Data Availability

UMINT has been implemented in Python 3. The codes to reproduce the results are freely available at https://github.com/deeplearner87/UMINT. GitHub repository has been organized into three main directories—Preprocessing, Proposed and Benchmarking. The Preprocessing directory (https://github.com/deeplearner87/UMINT/tree/main/Preprocessing) contains codes (R scripts and IPython notebooks) for preprocessing the datasets used in this study. The Proposed directory (https://github.com/deeplearner87/UMINT/tree/main/Proposed) contains the script for the proposed method umint.py and notebooks for the pipeline executed on various datasets. Notebooks corresponding to the comparative analysis made in this work are contained in the directory Benchmarking (https://github.com/deeplearner87/UMINT/tree/main/Benchmarking). The preprocessed datasets used in this work can be downloaded from https://doi.org/10.5281/zenodo.7723340. UMINT can be executed on any standard computing platform with at least 8 GB RAM on a Windows/Linux/CentOS platform with python 3.7+ installed in it.
