# Supplementary material for: Unsupervised neural network for single cell Multi-omics INTegration (UMINT): an application to health and disease
Source: Front Mol Biosci. 2023 May 24;10:1184748. doi: 10.3389/fmolb.2023.1184748 (PMC10244650; doi:10.3389/fmolb.2023.1184748)
Supplement: Supplementary file 1 [file DataSheet1.pdf]

UMINT: Unsupervised neural network for single cell Multi-omics INTegration

Supplementary Material

Chayan Maitra[1,†], Dibyendu B. Seal[2,†], Vivek Das[3,*], and Rajat K. De[1,*]

[1]*Machine Intelligence Unit, Indian Statistical Institute, 203 Barrackpore Trunk Road, Kolkata 700108, India.*
[2]*Tatras Data Services Pvt. Ltd., E64, Vasant Marg, Vasant Vihar, New Delhi 110057, India.*
[3]*Novo Nordisk A/S, Novo Nordisk Park 1, 2760 Maløv, Denmark.*
[†]*Authors contributed equally*
[*]*Corresponding authors: Vivek Das, vivekdas.0687@gmail.com, Rajat K. De, rajat@isical.ac.in*

May 9, 2023

S1 Performance of UMINT on single-cell multi-omics datasets as compared to Autoencoder (AE)-based methods

Table S1 shows the Median Correlation Coefficient (MCC) values and p-values for Overall Reconstruction Scores(ORSs) obtained using UMINT and the AE-based methods on *cbmc8k*, $MALT10k$ and *bmcite*30*k* datasets.

Table S1: Median Correlation Coefficient (MCC) values and p-values for Overall Reconstruction Scores(ORSs) obtained using UMINT and the AE-based methods on *cbmc8k*, $MALT10k$ and *bmcite*30*k* datasets.

	MCC				p value		
	**UMINT**	**AE**	**DAE**	**SAE**	**UMINT vs. AE**	**UMINT vs. DAE**	**UMINT vs. SAE**
**cbmc8k**	0.929	0.817	0.817	0.603	0.0001	0.0001	0.0001
**MALT10k**	0.92	0.832	0.825	0.635	0.0001	0.0001	0.0001
**bmcite30k**	0.927	0.805	0.805	0.484	0.0001	0.0001	0.0001

Table S2 shows the Median ARI (MARI), Median FMI (MFMI) scores and the corresponding p-values obtained using UMINT and the AE-based models on *cbmc8k*, $MALT10k$ and *bmcite*30*k* for both hierarchical and k-means clustering.

S2 Performance of UMINT on bulk multi-omics data

As a further extension to this work, we have also assessed UMINT for its integration performance on bulk expression datasets. TCGA multi-omics data for Liver Hepatocellular Carcinoma (LIHC) from TCGA portal (now moved to Genomic Data Commons `https://gdc.cancer.gov/`), have been used for this purpose. The dataset contains three omics layers - DNA methylation (DNAm), Copy Number Variation (CNV) and gene expression (RNA-seq). Pre-processed datasets collected from `http://doi.org/10.5281/zenodo.3712496`, contains 404 paired samples out of which 359 are cancer and 45 are normal.

To assess the effectiveness of UMINT on bulk multi-omics data, we have first compared the reconstruction and classification performance of UMINT against that obtained using a standard AE. As shown in Figure S1a, ORS for reconstruction of DNAm, CNV and RNA-seq obtained using UMINT has been found to be better than that obtained using AE ($UMINT_{MCC} = 0.397$, $AE_{MCC} = 0.336$, p-value = 0.009). For assessing the classification

Table S2: Median ARI (MARI), Median FMI (MFMI) scores and p-values for clustering performance of UMINT and AE-based models on *cbmc8k*, *MALT10k* and *bmcite30k* datasets for both hierarchical and k-means clustering.

**Clustering performance (Hierarchical)**							
	**MARI**				**p value**		
	**UMINT**	**AE**	**DAE**	**SAE**	**UMINT vs. AE**	**UMINT vs. DAE**	**UMINT vs. SAE**
**cbmc8k**	0.681	0.57	0.557	0.3	0.00018	0.00018	0.00018
**MALT10k**	0.592	0.61	0.613	0.14	0.001	0.03763	0.00018
**bmcite30k**	0.826	0.66	0.664	0.08	0.00058	0.00018	0.00018
	**MFMI**				**p value**		
	**UMINT**	**AE**	**DAE**	**SAE**	**UMINT vs. AE**	**UMINT vs. DAE**	**UMINT vs. SAE**
**cbmc8k**	0.761	0.67	0.653	0.44	0.00018	0.00018	0.00018
**MALT10k**	0.681	0.7	0.699	0.3	0.00076	0.02574	0.00018
**bmcite30k**	0.845	0.7	0.699	0.16	0.00058	0.00018	0.00018
**Clustering performance (k-means)**							
	**MARI**				**p value**		
	**UMINT**	**AE**	**DAE**	**SAE**	**UMINT vs. AE**	**UMINT vs. DAE**	**UMINT vs. SAE**
**cbmc8k**	0.656	0.57	0.596	0.3	0.00728	0.01725	0.00018
**MALT10k**	0.644	0.61	0.608	0.14	0.21229	0.04515	0.00018
**bmcite30k**	0.775	0.63	0.661	0.06	0.00018	0.00018	0.00018
	**MFMI**				**p value**		
	**UMINT**	**AE**	**DAE**	**SAE**	**UMINT vs. AE**	**UMINT vs. DAE**	**UMINT vs. SAE**
**cbmc8k**	0.734	0.65	0.69	0.41	0.01132	0.01725	0.00018
**MALT10k**	0.71	0.7	0.698	0.28	0.42735	0.08897	0.00018
**bmcite30k**	0.801	0.67	0.697	0.16	0.00018	0.00018	0.00018

performance, we have first integrated three modalities, viz., DNAm, CNV and RNA-seq, and extracted a low-dimensional embedding using UMINT. This low-dimensional embedding has been divided into training and test data following a 80 : 20 ratio. A multi-layer perceptron classifier has then been trained on 80% training data and its performance measured on the remaining 20% test data. The overall classification accuracy, recall and F1-scores achieved using the UMINT-embedding on the held-out test dataset, have been higher than that achieved by AE, as shown in Figure S1b.

In our earlier work, we had proposed a deep denoising autoencoder and multi-layer perceptron (DDAE-MLP)-based method for integrating such bulk multi-omics datasets, though the question addressed earlier was whether we could predict one omics data (gene expression) from the other two (DNAm and CNV), in case the mRNA data is of degraded quality or not available at all. As already stated earlier, UMINT can efficiently integrate variable number of omics layers. In order to explore this further, in this work, we have tried to find out if UMINT can capture better feature variability by integrating all three omics layers (DNAm, CNV and RNA-seq) simultaneously. Additionally, since MOFA+ can integrate both single cell and bulk multi-omics data, we have considered MOFA+ while comparing the classification performance of UMINT as well. With a similar $80-20$ train-test split, UMINT has been observed to perform competitively against MOFA+, as measured by classification accuracy, precision, recall and F1-scores. Figure S2 shows the results for this part of evaluation.

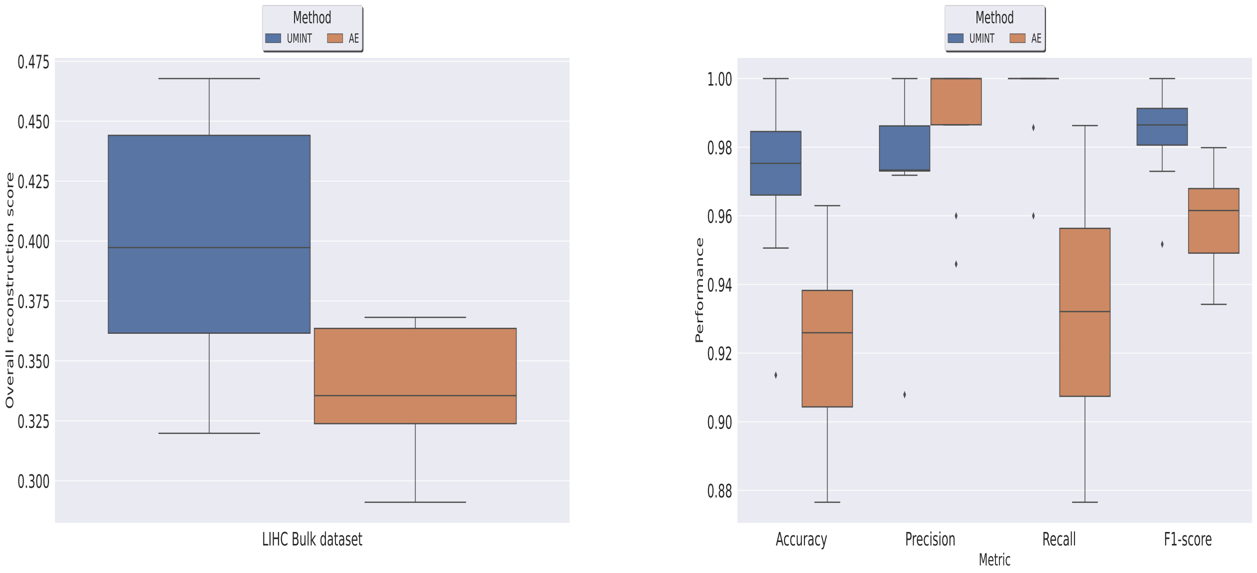

(a) Reconstruction

(b) Classification of Tumour/Normal Samples

Figure S1: (a) shows the performance of UMINT compared against an autoencoder (AE) network with respect to the overall reconstruction of DNA methylation (DNAm), Copy Number Variation (CNV) and RNA-seq expressions for TCGA LIHC dataset; (b) shows the performance of UMINT compared against an AE with respect to classification of tumour and normal samples on TCGA LIHC dataset.

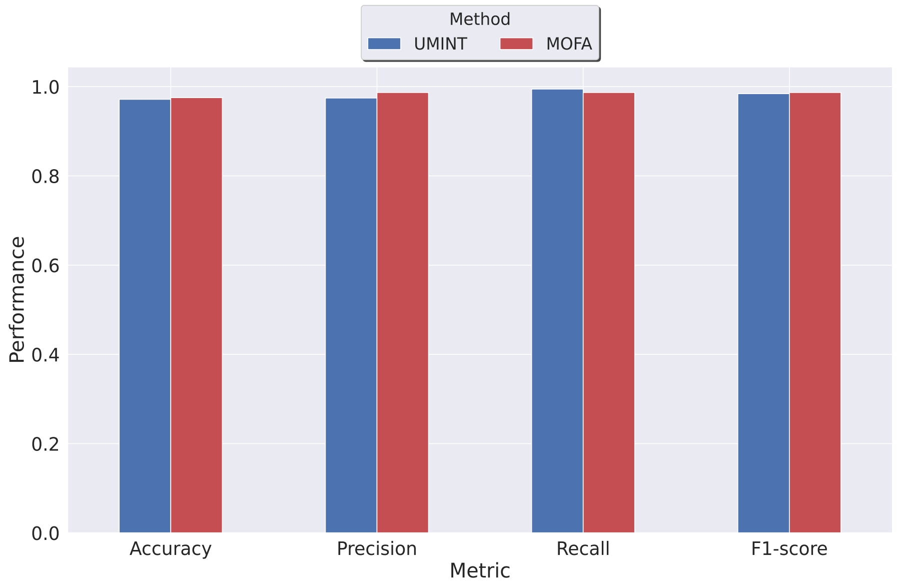

Figure S2: Classification performance achieved on UMINT-generated low-dimensional embedding compared against that obtained on MOFA+-generated embedding for TCGA LIHC dataset.
